# Transcriptomic and Proteomic Insights into 6PPD/6PPD-Q Induced Oxidative Stress in Black-Spotted Frogs

**DOI:** 10.3390/antiox14081019

**Published:** 2025-08-20

**Authors:** Wenhui Sun, Bingyi Wang, Sihan Zhang, Zhiquan Liu, Yinan Zhang, Hangjun Zhang

**Affiliations:** Zhejiang Provincial Key Laboratory of Wetland Intelligent Monitoring and Ecological Restoration, School of Engineering, Hangzhou Normal University, Hangzhou 310018, China; 2023111010066@stu.hznu.edu.cn (W.S.); 2023111010069@stu.hznu.edu.cn (B.W.); 2023213303028@stu.hznu.edu.cn (S.Z.); 20250042@hznu.edu.cn (Y.Z.); 20080099@hznu.edu.cn (H.Z.)

**Keywords:** 6PPD, 6PPD-Q, *Pelophylax nigromaculatus*, transcriptomic, proteomic

## Abstract

N-(1,3-dimethylbutyl)-N′-phenyl-p-phenylenediamine (6PPD) and its oxidation product 6PPD-quinone (6PPD-Q) can have lethal effects on aquatic organisms, interfering with gene expression and protein content in aquatic animals. In this study, we performed proteomics and transcriptomics analyses on the livers of black-spotted frogs exposed to 6PPD and 6PPD-Q. The results showed that 6PPD and 6PPD-Q can cause oxidative stress in the liver, significantly reducing catalase (CAT) and glutathione peroxidase (GSH-Px) levels, with 6PPD-Q having a more significant toxic effect. Through transcriptomics and proteomics analysis, this study identified oxidative stress and immune defense pathways. In this study, the liver of the black-spotted frog provided some molecular insights into the toxicity of 6PPD and 6PPD-Q. Nonetheless, additional investigations are required to gain a clearer comprehension of the possible mechanisms that drive how aquatic organisms react to the toxic effects of 6PPD and 6PPD-Q.

## 1. Introduction

With the acceleration of global urbanization, the popularity of automobiles continues to increase, and the frequency of tire use has also increased accordingly. Tire production worldwide can reach as high as 2 billion units. N-(1,3-dimethylbutyl)-N′-phenyl-p-phenylenediamine (6PPD) serves as a widely used tire additive, functioning as an anti-ozone component or antioxidant to boost tire sturdiness and operational lifespan [1]. 6PPD reacts with ozone in water to form 6PPD-quinone (6PPD-Q), a compound linked to high mortality in migratory fish species [2], illustrating urban runoff death syndrome.

6PPD and 6PPD-Q are frequently detected in aquatic settings. A case in point is urban runoff. 6PPD-Q concentrations reached as high as 4100–6100 ng/L and 800–19,000 ng/L in the US cities of Los Angeles and Seattle [3], while concentrations in Hong Kong were 210–2430 ng/L [3]. In 57% of rainwater runoff, 6PPD-Q was found, and its levels varied between 86 and 1400 ng/L [4]. In wastewater, the concentration of 6PPD (300–4300 ng/L) is higher than that of 6PPD-Q (52–105 ng/L) [5]. These frequent detection results have sparked widespread concern about the poisonous impacts exerted by 6PPD and 6PPD-Q.

Beyond deadly impacts, 6PPD and 6PPD-Q might induce developmental toxicity, behavioral toxicity, oxidative stress, and lipid metabolism issues in water-dwelling creatures [6,7,8]. For example, exposing zebrafish to 25 μg/L concentrations of both 6PPD and 6PPD-Q can induce physical deformities and morphological changes in the eyes of the test subjects [9]. Adult zebrafish subjected to ecologically relevant dosages (20 μg/L) of 6PPD and 6PPD-Q for 28 days showed histological evidence of more significant hepatic vacuolation and lipid accumulation induced by 6PPD than by 6PPD-Q, suggesting that 6PPD has stronger hepatotoxicity [10]. Therefore, subsequent investigations should be conducted to contrast the toxicological properties of 6PPD and 6PPD-Q. However, various research studies indicate that 6PPD-Q’s fatal impacts display marked species-specific toxicity. To illustrate, *Salvelinus leucomaenis pluvius* exhibits acute sensitivity to 6PPD-Q, with a 24 h median lethal concentration (LC_50_) of 510 ng/L, whereas it shows no lethal toxicity to *Salvelinus curilus* and *Oncorhynchus masou masou* [11]. Recent studies have revealed that 6PPD-Q can induce molecular damage, such as DNA adduct formation and ferroptosis [12,13]. Therefore, more aquatic organisms are needed to verify the toxicity of 6PPD and 6PPD-Q, including amphibians.

Amphibians frequently act as bioindicator species for assessing ecosystem well-being. Nevertheless, global amphibian numbers are now undergoing a notable drop, and environmental contamination stands as the primary cause of this population decrease, endangering the existence of 19% of amphibian types [14]. The black-spotted frog (*Pelophylax nigromaculatus*) ranks among the amphibian species with the broadest distribution in China. It not only plays an important ecological role in aquatic ecosystems but also has significant economic value [15,16]. The reproduction and development stages of this species are dependent on an aquatic environment, so pollutants in the water have a direct impact on its growth and development, endocrine regulation, and circulatory system [17,18]. Based on these characteristics, the black-spotted frog has become a model species for toxicological research and has unique advantages in assessing the biological toxicity effects of water pollutants [19,20]. It is important to emphasize that its ecological characteristics also make it an ideal indicator organism for assessing the biological effects of pollutants such as 6PPD and 6PPD-Q in urban runoff.

With the development of transcriptomics and proteomics, the mechanisms underlying the responses of aquatic animals to 6PPD and 6PPD-Q stress have been investigated. From a molecular perspective, the liver constructs a complete molecular regulatory network from gene expression regulation to protein function execution, thereby participating in the detoxification of 6PPD and 6PPD-Q [21]. Transcriptomics can comprehensively reveal dynamic changes in gene expression profiles through high-throughput sequencing, thereby systematically analyzing the activation of signal transduction pathways triggered by exposure to pollutants [22,23]. Proteomics uses high-resolution mass spectrometry technology to quantitatively analyze changes in protein expression levels in liver tissue, thereby elucidating the key functional protein networks affected by pollutants [24,25]. Thus, combining transcriptomic and proteomic datasets is required to delve deeper into shifts in genes and proteins within frog livers and to grasp the molecular response ways of frogs when exposed to 6PPD and 6PPD-Q.

Within the present research, frogs received 21-day exposure to 6PPD and 6PPD-Q at environmental levels, and we assessed the extent of liver damage. We then conducted transcriptomics and proteomics analyses to investigate the potential molecular mechanisms involved. Our investigation into how 6PPD and 6PPD-Q affect the liver of the black-spotted frog has enhanced our grasp of the possible dangers to amphibian well-being and ecological stability.

## 2. Materials and Methods

### 2.1. Reagents

6PPD (CAS: 793-24-8; purity > 98%) was purchased from TCI (Shanghai, China). 6PPD-Q was purchased from J&K Scientific (Beijing, China). Dimethyl sulfoxide (DMSO; purity > 99.9%) was purchased from Yeasen Biotechnology Co., Ltd. (Shanghai, China). The remaining chemicals were all of analytical or chromatographic purity.

### 2.2. Experimental Design

The rearing of *Pelophylax nigromaculatus* conformed to our pre-established procedures [26]. Male creatures showed steadier hormonal patterns compared to females, enabling a more accurate depiction of trial outcomes [27]. As a result, we chose male frogs as the test subjects. After a period of acclimatization to the culturing environment, we arbitrarily split two-year-old male frogs into five groups, with three repetitions per group: the control group, along with 1 μg/L 6PPD, 10 μg/L 6PPD, 1 μg/L 6PPD-Q, and 10 μg/L 6PPD-Q. The concentration selection is based on the actual concentrations of 6PPD and 6PPD-Q in aquatic environments [2,28]. We administered a 0.01% DMSO dose to each frog across all groups. Each of the 15 tanks housed 20 frogs. We replaced two liters of exposure solution daily over the entire test period to sustain 6PPD and 6PPD-Q levels, with temperature set at 20 ± 1 °C, pH at 6.5 ± 0.5, and dissolved oxygen level at 7 ± 1 mg/L.

After a 21-day test phase, we put down the test subjects by cutting the spinal cord and later performed dissections to collect tissues. The liver specimens were maintained intact and stored at −80 °C for later analysis. To reduce differences between individuals, liver specimens from three frogs were combined to form one biological repeat. These grouped samples were used to evaluate all endpoints. We collected three samples for biochemical assays, and we used the remaining samples for omics analysis. All animal experiments adhered to the ethical guidelines established by the Association of Laboratory Animal Sciences (No. HSD20240101, Hangzhou Normal University).

### 2.3. Biochemical Assays

To mitigate instrument-related variability, each sample underwent triplicate analysis. Levels of catalase (CAT) and glutathione peroxidase (GSH-PX) in the samples were quantified with commercial test kits from Nanjing Jiancheng Bioengineering Institute in Nanjing, China, adhering to the maker’s instructions.

### 2.4. Molecular Docking

We simulated CAT and GSH-Px proteins using transcriptome data and the SWISS-MODEL approach (https://swissmodel.expasy.org/ (accessed on 1 July 2025)). Subsequently, PlayMolecule (https://www.playmolecule.com/ (accessed on 1 July 2025)) was used to predict the binding pocket of the two proteins. Download the 3D structures of 6PPD and 6PPD-Q from PubChem (https://pubchem.ncbi.nlm.nih.gov/ (accessed on 1 July 2025)). We used AutoDock Tools (1.5.7) for pre-processing of pollutant and protein files. Then, we used AutoDock Vina for molecular docking. To ensure the reliability of the docking results, each docking was carried out in triplicate, and only binding poses that consistently showed the lowest binding energy were considered. The final docking conformations were visualized using Discovery Studio Visualizer 2021 Client (San Diego, CA, USA).

### 2.5. Transcriptome

Total RNA was isolated from the specimens using TRIzol reagent (Invitrogen, Carlsbad, CA, USA). Subsequently, mRNA was purified from the total RNA extract by Oligo(dT) magnetic beads and then reverse-transcribed into cDNA. After that, the cDNA-carrying adaptors underwent purification and size selection to form the final library. The library thus built was sequenced in paired ends with the Illumina Novaeq 6000 (LC Bio, Hangzhou, China). The sequencing reads were mapped to the reference genome via Hisat2, and expression levels were quantified with RSEM to identify the expression levels of different genes. Finally, analysis of differential expression was carried out with the R program Version 4.3.0 and the DESeq2 package to determine genes that exhibited significant changes under varying conditions. Other parts were analyzed on the online platform of Omicstudio Cloud Platform version 3.5.7 (https://www.omicstudio.cn/doc (accessed on 1 July 2025)). Genes with |log2 fold change| ≥ 1 and adjusted *p*-value (FDR) < 0.05 were considered differentially expressed. Enrichment analyses were performed using the Kyoto Encyclopedia of Genes and Genomes (KEGG) and Gene Ontology (GO) databases, with significance defined as *p* < 0.05 after Benjamini–Hochberg correction.

### 2.6. Proteome

The specimens were processed for total protein isolation with a protein extraction buffer, followed by enzymatic digestion with trypsin. The resulting peptide samples were labeled using TMT reagents (Thermofisher, Waltham, MA, USA). One-dimensional separation of the samples was performed using reversed-phase liquid chromatography, and the final fractions were determined based on peak shape and retention time. Subsequently, peptide analysis was conducted using liquid chromatography–tandem mass spectrometry. Protein identification was performed by aligning the sequencing data with a protein database using ProteomeDiscovererTM Software 2.4. The *t* test function in R was used to calculate the *p*-values for the significance of differences between samples, and the fold change (FC) between groups was also calculated. Other parts were analyzed on the online platform of Omicstudio Cloud Platform version 3.5.7 (https://www.omicstudio.cn/doc, accessed on 1 July 2025). Proteins showing a fold change ≥ 1.2 or ≤ 0.83 and an adjusted *p*-value < 0.05 were classified as differentially expressed. Enrichment analyses were performed using the KEGG and GO databases, with significance defined as *p* < 0.05 after Benjamini–Hochberg correction.

### 2.7. Statistical Analysis

Results are shown as average ± standard error of the mean. The Kolmogorov–Smirnov test was applied to check normality, and Levene’s test was used to examine variance homogeneity before conducting analyses. Comparisons between groups were analyzed via one-way ANOVA, followed by Tukey’s multiple range test (IBM SPSS Statistics 25, New York, NY, USA). For non-normal data spread, the Kruskal–Wallis test was employed. A *p*-value below 0.05 was set to indicate statistical significance.

## 3. Results

### 3.1. 6PPD and 6PPD-Q Induced Oxidative Stress in the Liver

**Figure 1:**
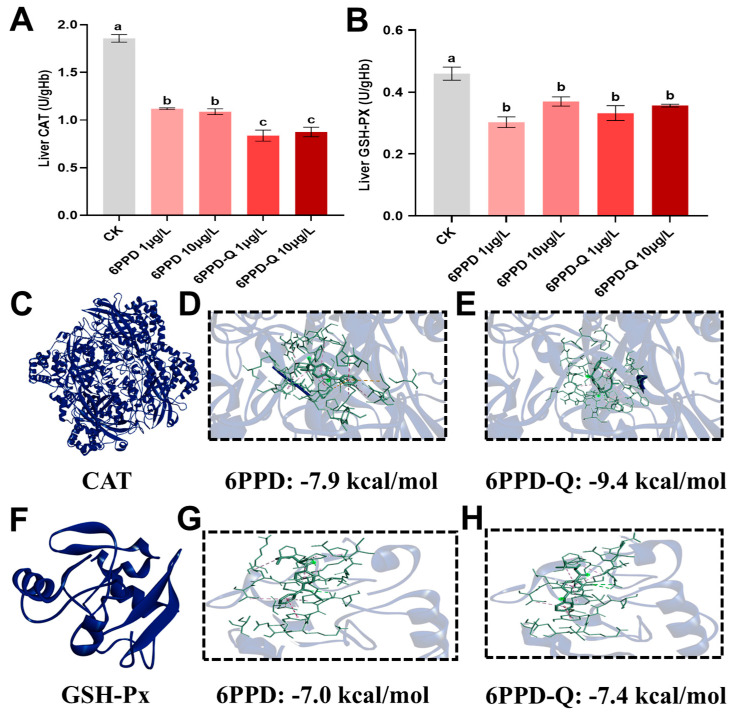
Being exposed to 6PPD and 6PPD-Q caused changes in the oxidative stress index levels: (**A**) CAT and (**B**) GSH-PX. Data are presented as mean ± standard error of the mean (n = 3). Superscript letters represent significant differences between groups (*p* < 0.05). Simulated proteins of (**C**) CAT and (**F**) GSH-Px in frogs; bonding modes of (**D**) 6PPD and (E) 6PPD-Q with CAT; bonding modes of (**G**) 6PPD and (**H**) 6PPD-Q with GSH-Px.

After being exposed to 6PPD and 6PPD-Q, the CAT content in frog livers dropped notably (*p* < 0.05, Figure 1A). When set against the CK group, being exposed to 1 and 10 μg/L 6PPD reduced CAT activity by 39.74% and 41.41%, respectively; exposure to the same concentrations of 6PPD-Q reduced CAT activity by 54.93% and 52.90%, respectively. The decrease in CAT activity in the livers of frogs being subjected to 6PPD-Q was significantly greater than the group exposed to 6PPD, suggesting that 6PPD-Q has a stronger oxidative stress toxicity effect. The docking scores of 6PPD and 6PPD-Q with CAT stood at −7.9 and −9.4 kcal/mol (Figure 1C–E). Outcomes from molecular docking indicated that 6PPD-Q bound to CAT far more tightly than 6PPD did.

After exposure to 6PPD and 6PPD-Q, the GSH-Px content in frog livers fell markedly (*p* < 0.05, Figure 1B). Compared with the CK group, exposure to 1 and 10 μg/L 6PPD reduced CAT activity by 34.10% and 19.53%, respectively; exposure to the same concentrations of 6PPD-Q reduced CAT activity by 27.75% and 22.43%, respectively. The docking scores of 6PPD and 6PPD-Q with GSH-Px were −7.0 and −7.4 kcal/mol (Figure 1F–H). 6PPD-Q also formed a tighter connection with GSH-Px.

### 3.2. Transcriptomic Analysis

#### 3.2.1. Analysis of Differentially Expressed Genes, GO, and KEGG Enrichment

**Figure 2:**
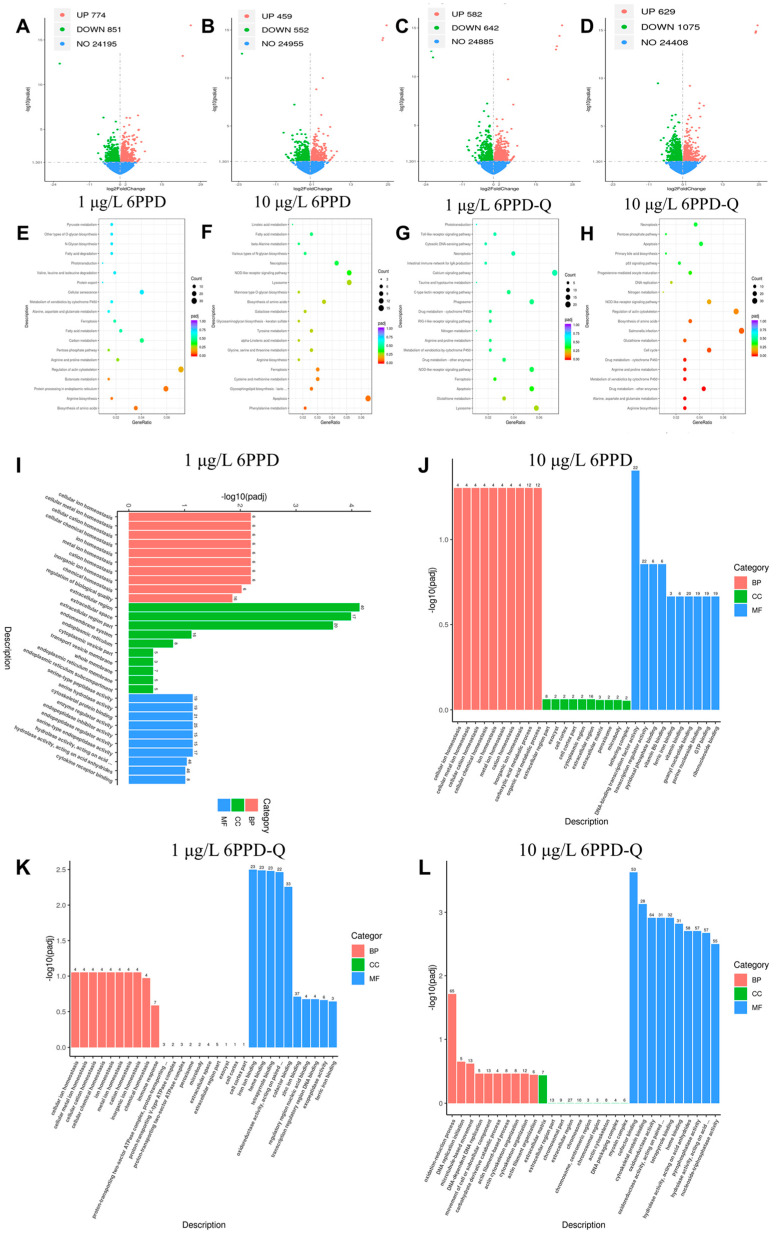
Analysis of DEGs distribution and enrichment. (**A**–**D**) Volcano plots of DEGs between the exposed group and the control group; (**E**–**H**) KEGG bubble plots of DEGs between the exposed group and the control group; (**I**–**L**) GO diagrams illustrating DEGs from the exposed versus control sets (encompassing biological processes (BPs), molecular functions (MFs), and cellular components (CCs)) across the three comparison sets.

For a deeper exploration of how black-spotted frogs molecularly respond to 6PPD and 6PPD-Q at environmental levels, transcriptomic tests were carried out on their liver tissues. In this study, 1625 (774 increased in expression and 851 decreased in expression) and 1011 (459 increased in expression and 552 decreased in expression) molecular features were identified as statistically significant differentially expressed genes (DEGs) in the 1 (Figure 2A) and 10 (Figure 2B) μg/L 6PPD exposure groups, respectively; 1224 (582 increased in expression and 642 decreased in expression) and 1704 (629 increased in expression and 1075 decreased in expression) molecular features were identified as statistically significant DEGs in the 1 (Figure 2C) and 10 (Figure 2D) μg/L 6PPD-Q exposure groups, respectively. Compared with the control set, the 10 μg/L 6PPD-Q group contained the greatest count of differentially expressed genes, signifying that liver RNA underwent notable changes post-exposure.

GO analysis revealed that in the 1 μg/L 6PPD exposure group, DEGs primarily participated in biological functions, such as cellular homeostasis maintenance, extracellular and membrane-related processes, and protein metabolism (Figure 2E). In the 10 μg/L 6PPD group, DEGs were significantly enriched in cellular homeostasis maintenance, cellular metabolism, cellular structure and interaction, and protein metabolism and modification (Figure 2F). In the 1 μg/L 6PPD-Q group, DEGs primarily involved themselves in cellular homeostasis maintenance, immune response, cellular structure and interaction, and redox reactions and gene expression regulation, with significant enrichment (Figure 2G). In the 10 μg/L 6PPD-Q group, DEGs were significantly enriched in redox reactions, DNA replication, cytoskeletal organization, extracellular matrix-related processes, and processes involving multiple enzyme activities and binding functions (Figure 2H). GO analysis revealed that when frogs are exposed to 6PPD and 6PPD-Q, their liver tissues primarily sustain cellular stability and react to harm via processes like strengthened ion transmembrane movement, redox reactions, DNA repair and replication, cytoskeletal remodeling, extracellular matrix metabolism, and metabolic regulation.

Through KEGG pathway analysis, this study found that multiple key metabolic and signaling pathways in the liver underwent significant changes following exposure to 6PPD and 6PPD-Q. Of these, routes like glutathione metabolism, cell apoptosis, arginine and proline metabolism, and xenobiotic processing via cytochrome P450 showed marked enrichment in every group. This points to their key function in how the liver reacts to harm from exposure (Figure 2I–L). Compared with 6PPD-Q, 6PPD can induce ferroptosis accompanied by a decrease in glutathione content, and this process is significantly correlated with hepatotoxicity [13]. This matches the findings obtained in the present research. The shared enrichment of these routes indicates that the liver mainly copes with harm after exposure via processes like boosting antioxidant protection systems, regulating cell death processes, activating immune and inflammatory responses, maintaining cell structure and function, and metabolizing and detoxifying processes. This suggests that the liver, upon exposure, activates and enhances these key pathways to maintain cellular homeostasis, mitigate oxidative damage, regulate immune and inflammatory responses, and promote the repair of cellular structure and function, thereby enhancing the liver’s tolerance and recovery capacity in response to exposure-induced damage. In addition, representative DEGs are summarized in Table S1. Most of these genes are closely linked to oxidative stress regulation and immune-related pathways, further supporting the enrichment of glutathione metabolism and ferroptosis.

#### 3.2.2. Analysis of Weighted Gene Co-Expression Network

**Figure 3:**
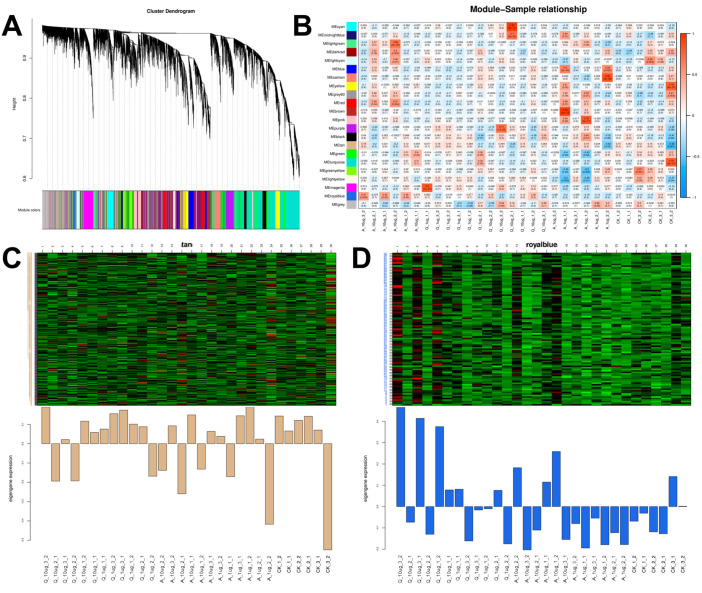
WGCNA in frogs after 6PPD and 6PPD-Q exposure. (**A**) Module hierarchical clustering; (**B**) heat map showing correlations between samples and modules; (**C**) MEtan and (**D**) MEroyalblue gene clustering heat map.

Weighted gene co-expression network analysis (WGCNA) was employed to investigate the correlation between various physiological traits and identify pivotal genes within the liver of the black-spotted frog. Transcripts with FPKM values less than 1 were excluded from the analysis. In WGCNA, the connection between these two modules was characterized as gene clusters with high correlation, and genes in the same cluster showed tight associations. In all, 22 modules were recognized (Figure 3A). Among all modules, the MEtan and MEroyalblue modules showed a strong correlation with all samples (Figure 3B), suggesting that these modules may be the gene cluster with the most significant gene expression changes following exposure to 6PPD and 6PPD-Q. Specifically, in the MEtan module, the control group gene cluster showed a high expression pattern, but the expression level declined subsequent to exposure to 6PPD and 6PPD-Q (Figure 3C). In contrast, in the MEroyalblue module, the gene cluster showed low expression within the control set while showing high expression in the exposed set (Figure 3D). Within each of the two modules, the count of gene cluster changes in the 6PPD-Q-exposed group exceeded that in the 6PPD group.

### 3.3. Proteomic Analysis

#### 3.3.1. Differentially Expressed Proteins and Subcellular Localization Prediction

**Figure 4:**
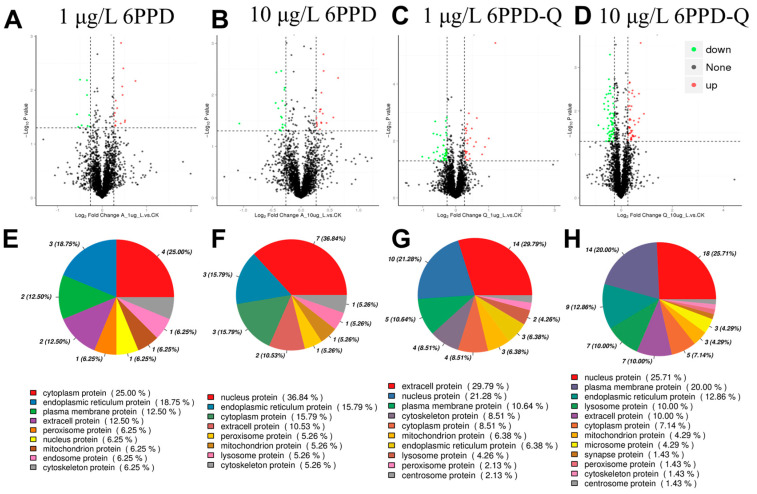
Differentially expressed proteins and subcellular localization prediction. (**A**–**D**) Volcano plots of DEPs between the exposed group and the control group; (**E**–**H**) DEPs subcellular localization analysis.

In order to clarify how the liver of the black-spotted frog reacts to 6PPD and 6PPD-Q exposure, this research applied proteomic methods to study proteins in the frog’s liver, aiming to detect proteins with differential expression (DEPs) post-exposure. In the 1 (Figure 4A) and 10 (Figure 4B) μg/L 6PPD exposure group, we detected 24 DEPs (15 with increased expression and 9 with decreased expression) and 34 DEPs (17 with increased expression and 17 with decreased expression), respectively. In the 1 (Figure 4C) and 10 (Figure 4D) μg/L 6PPD-Q exposure group, we detected 70 DEPs (29 with increased expression and 41 with decreased expression) and 34 DEPs (15 with increased expression and 19 with decreased expression), respectively. In the four exposure groups, most DEPs were concentrated in the cytoplasm (Figure 4E–H). When frogs were exposed to 1 μg/L 6PPD, the subcellular positioning of DEPs was mainly within the cytoplasm and endoplasmic reticulum (Figure 4E). Low concentrations of 6PPD may induce the release of inflammatory cytokines by interfering with cytoplasmic metabolic pathways and endoplasmic reticulum protein folding functions, thereby causing liver barrier dysfunction [29]. Within the 10 μg/L 6PPD group, DEPs were mainly distributed in the nucleus and endoplasmic reticulum (Figure 4F). High doses of 6PPD may exacerbate hepatotoxicity by regulating nuclear gene transcription [30]. Upon exposure to 1 μg/L 6PPD-Q, DEPs significantly aggregated in the extracellular region and the nucleus (Figure 4G). Low concentrations of 6PPD-Q may initiate toxic effects through extracellular signaling or nuclear receptor-mediated pathways [31]. Within the 10 μg/L 6PPD-Q group, DEPs were primarily localized in the nucleus and plasma membrane (Figure 4H). High doses of 6PPD-Q may simultaneously affect nuclear gene expression regulation and cell membrane integrity, exacerbating liver damage [32,33].

#### 3.3.2. Analysis of GO and KEGG Enrichment

**Figure 5:**
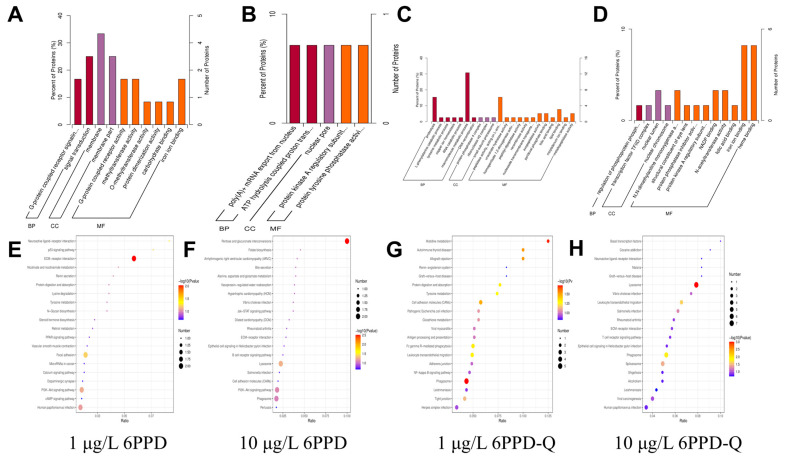
Analysis of DEPs distribution and enrichment. (**A**–**D**) GO diagrams illustrating DEPs from the exposed versus control sets (encompassing biological processes (BPs), molecular functions (MFs), and cellular components (CCs)) across the three comparison sets; (**E**–**H**) KEGG bubble plots of DEGs between the exposed group and the control group.

The GO pathways enriched in the 1 μg/L 6PPD group covered multiple aspects, including cell signaling, substance transport, metabolic regulation, cell structure composition, and intermolecular interactions (Figure 5A). The 10 μg/L 6PPD group showed significant enrichment in biological processes and functions such as gene expression regulation, energy metabolism, substance transport, cell signal transduction, and protein modification (Figure 5B). In the 1 μg/L 6PPD-Q group, significant changes occurred in pathways related to protein metabolism, substance decomposition, DNA repair and recombination, cell signal transduction, and intracellular and extracellular substance transport (Figure 5C). In the 10 μg/L 6PPD-Q group, DEPs were significantly enriched in pathways related to gene expression regulation, protein modification, intracellular metabolism, and cell signal transduction (Figure 5D).

Through KEGG pathway analysis, the present research identified extracellular matrix-receptor interactions and PI3K-Akt signaling pathways as major metabolic routes in the 6PPD-exposed group. In both pathways, DEPs showed significantly greater enrichment (Figure 5E,F). In the 6PPD-Q exposure group, KEGG pathway analysis revealed that extracellular matrix-receptor interactions and phagocytosis pathways were key metabolic pathways. In both pathways, DEPs showed a significantly higher enrichment level (Figure 5G,H). ECM-receptor interactions and phagocytosis pathways showed significant enrichment in different exposure groups, suggesting that these pathways play a key role in the response to 6PPD and 6PPD-Q exposure. The extracellular matrix (ECM) forms an intricate, active network structure made up of large molecular compounds released by cells into the extracellular area. It consists of the interstitial matrix and basement membrane and accounts for more than one-third of the body’s mass. The ECM and immune cells are interdependent and can serve as guides for the movement and positioning of immune cells [34,35]. The phagocytic pathway may be involved in cellular immune defense and pathogen clearance [36]. Similarly, key DEPs are listed in Table S2, the majority of which are involved in oxidative stress responses and immune processes, consistent with the observed alterations in metabolic regulation and antigen-processing pathways.

## 4. Discussion

Due to the widespread distribution of 6PPD and 6PPD-Q in aquatic environments, recent studies have considered potential biological effects associated with exposure [37]. However, the majority of research has not probed into possible molecular mechanisms of downstream pathways when exposed to 6PPD and 6PPD-Q. Therefore, transcriptomics and proteomics analyses are useful in pointing us toward more accurate mechanism studies. On the whole, alterations in the expression of genes and proteins linked to two physiological processes were detected: oxidative stress and immune defense. Notably, the transcriptome revealed broader changes across multiple pathways, whereas the proteome reflected a more restricted set of protein-level alterations, which is likely attributable to post-transcriptional and translational regulation.

### 4.1. Oxidative Stress

Oxidative stress may be induced by increased amounts of reactive oxygen species (ROS), also known as free radicals, in the organism. These extremely reactive substances have the potential to harm cellular elements, such as DNA, proteins, and lipids. Should the generation of free radicals go beyond the organism’s antioxidant protection mechanisms, oxidative stress will take place [38,39]. Exposure to environmental pollutants like 6PPD and 6PPD-Q can exacerbate the production of free radicals, thereby contributing to oxidative stress [40]. Hydrogen peroxide, a byproduct of metabolic processes, can induce oxidative stress and cellular damage if not promptly eliminated [41]. CAT, through the swift degradation of hydrogen peroxide, averts its buildup, thus fulfilling a vital function in upholding the cell’s redox equilibrium [42]. GSH-Px, on the other hand, is an enzyme that promotes the glutathione (GSH)—mediated reduction of hydrogen peroxide [43]. GSH, a crucial tripeptide, possesses reductive and nucleophilic properties, safeguarding cells from attack by reactive oxygen species such as hydrogen peroxide. GSH-Px maintains intracellular glutathione levels by converting hydrogen peroxide into water and glutathione, thereby protecting cells from oxidative damage [44,45]. Following exposure to 6PPD and 6PPD-Q, the levels of CAT and GSH-Px in frog livers declined remarkably, which implies that 6PPD and 6PPD-Q possess oxidative stress toxicity.

Earlier research has employed omics techniques to examine and uncover the stress reaction mechanisms of zebrafish subjected to 6PPD and 6PPD-Q stressors [46]. The findings show that when under 6PPD and 6PPD-Q strain, zebrafish change the ways their liver genes are expressed. These findings suggest that aquatic organisms can dynamically regulate gene and protein expression to respond to 6PPD- and 6PPD-Q-induced toxicity and also indicate that gene and protein expression regulatory mechanisms play a key role in zebrafish antagonizing liver toxicity.

Transcriptomics and proteomics results indicate that the liver responds to damage and stress caused by exposure to 6PPD and 6PPD-Q by activating and enhancing a series of complex cellular and molecular mechanisms, including maintaining ion homeostasis, enhancing antioxidant defense, strengthening DNA repair and replication, remodeling the cytoskeleton, regulating extracellular matrix metabolism, finely regulating the cell death process, and comprehensively regulating metabolic activities. The synergistic action of these mechanisms helps the liver maintain cellular homeostasis, reduce oxidative damage, repair damaged cellular structures and functions, and promote tissue recovery and regeneration, thereby enhancing the liver’s tolerance and adaptability to post-exposure damage and ensuring the liver’s overall physiological function and health status after exposure. In this study, we found that glutathione metabolism was found to be involved in the defense against oxidative stress. Studies show that the glutathione metabolic route has a key part in antioxidant activity [47]. Glutathione is an antioxidant commonly found in aquatic animals. It can eliminate reactive oxygen species, help metabolize peroxides and heavy metals, and prevent harmful substances from damaging cells and DNA. It is an indicator of oxidative stress [48,49]. 6PPD and 6PPD-Q inhibited the glutathione metabolic pathway in frog liver, leading to oxidative stress.

By integrating multi-omics evidence, we can propose a working model: exposure to 6PPD and 6PPD-Q initially perturbs redox homeostasis, resulting in decreased CAT and GSH-Px activities and inhibition of glutathione metabolism. These oxidative disturbances trigger transcriptomic responses in glutathione metabolism, xenobiotic metabolism, apoptosis, and ferroptosis, while proteomic alterations highlight ECM–receptor interactions, PI3K-Akt signaling, and phagocytosis. In summary, these findings suggest that oxidative stress and post-transcriptional/translational regulation underlie the observed enzyme changes, which then propagate into broader molecular and cellular responses.

### 4.2. Immune Defense

Since 6PPD and 6PPD-Q are emerging environmental pollutants, they are likely to affect the immune systems of aquatic organisms [50]. In this study, many immune-related pathways, including ECM-receptor interactions, PI3K-Akt signaling, glutathione metabolism, and antigen processing/presentation, were significantly altered in frog livers in response to exposure to 6PPD and 6PPD-Q [51,52,53]. Among the aforementioned pathways of change, glutathione metabolism and antigen processing/presentation are particularly important because they play a crucial role in the immunotoxic response of aquatic organisms. Antigen-presenting cells are capable of phagocytosing, processing exogenous antigens, and presenting them to T lymphocytes, thereby activating a specific immune response [54]. Alterations in this pathway may indicate that, under exposure to 6PPD and 6PPD-Q, the ability of antigen-presenting cells to process and present antigens has changed, thereby affecting the activation of T lymphocytes and the initiation of the immune response. This may lead to a reduced ability of the body to recognize and clear pathogens or trigger abnormal immune reactions. However, in order to maintain normal immune system function, immune cells must be fully activated and differentiated, and glutathione plays a role in the full activation and differentiation of immune cells [55]. In the future, we must conduct additional straightforward investigations to confirm immune harm to frog livers following 6PPD and 6PPD-Q exposure, thereby backing our results. Nevertheless, these findings are based on pathway-level enrichment analyses, and we will require further functional assays to confirm direct immunotoxic effects in amphibians.

## 5. Conclusions

In this research, we employed omics-based analysis to explore how black-spotted frog livers react to stress from 6PPD and its metabolite 6PPD-Q at environmental levels. Our outcomes show that 6PPD and 6PPD-Q notably curb CAT and GSH-px expression in the liver, leading to oxidative stress. Through transcriptomic and proteomic analyses, this research uncovered how frog livers respond to stress from 6PPD and 6PPD-Q. Transcriptome analysis showed that DEGs in frog livers exposed to 6PPD and 6PPD-Q were enriched in pathways such as cellular homeostasis and metabolism. KEGG analysis showed that pathways such as glutathione metabolism were activated, and the liver responded to damage through multiple mechanisms. Proteome analysis showed that DEPs in the 6PPD exposure group were enriched in signaling transduction and metabolic regulation pathways, while the 6PPD-Q group significantly affected protein metabolism and DNA repair. The results from our study should offer fresh perspectives on how amphibians’ molecular response systems function when under 6PPD and 6PPD-Q stress. These molecular effects may influence amphibian population dynamics, though species differences and laboratory conditions limit broader extrapolation. In addition, the relatively small sample size and single-species focus may reduce generalizability. Future studies with larger cohorts and cross-species comparisons will be essential to validate and extend these findings.

## Data Availability

Data are contained within the article and Supplementary Materials.

## Supplementary Materials

The following supporting information can be downloaded at: https://www.mdpi.com/article/10.3390/antiox14081019/s1, Table S1. Differential expression of key transcription factors in the liver of the black-spotted frog was observed after exposure to 6PPD and 6PPD-Q. Table S2. Differential expression of key proteins in the liver of the black-spotted frog after exposure to 6PPD and 6PPD-Q.
