# Supplementary material for: Transcriptomic and Proteomic Insights into 6PPD/6PPD-Q Induced Oxidative Stress in Black-Spotted Frogs
Source: Antioxidants (Basel). 2025 Aug 20;14(8):1019. doi: 10.3390/antiox14081019 (PMC12382696; doi:10.3390/antiox14081019)
Supplement: Supplementary file 1 [file antioxidants-14-01019-s001.zip › antioxidants-3766676-supplementary.pdf]

Transcriptomic and Proteomic insights into 6PPD/6PPD-Q Induced Oxidative Stress in Black-Spotted Frogs

Wenhui Sun, Bingyi Wang, Wanze Ouyang, Zhiquan Liu [*], Yinan Zhang, Hangjun Zhang

Zhejiang Provincial Key Laboratory of Wetland Intelligent Monitoring and Ecological Restoration, School of Engineering, Hangzhou Normal University, Hangzhou, Zhejiang 310018, China

* Correspondence to: Zhiquan Liu, School of Engineering, Hangzhou Normal University, Hangzhou 310018, China

Email: zqliu@hznu.edu.cn

Table S1. Differential expression of key transcription factors in the liver of the black-spotted frog was observed after exposure to 6PPD and 6PPD-Q.

Transcription factor family	Gene ID	Symbol	Gene annotation
Glutathione	evm.TU.Chr8.788	GPX7	Enable catalase activity
	evm.TU.Chr4.3686	GCLC	The rate limiting enzyme for glutathione synthesis
	evm.TU.Chr9.679	GSTP1	Catalyze the binding of glutathione to toxic metabolites and promote their excretion
	evm.TU.Chr9.677	GSTA2	Cellular detoxification
	evm.TU.Chr4.3693	GSTA3	Cellular defense
Ferritin	evm.TU.Chr10.819	FTH1	Storage and metabolism of iron ions
SLC39A	evm.TU.Chr3.5828	SLC39A14	Non transferrin binding iron important transporter protein and key regulator of manganese homeostasis
GADD45	evm.TU.Chr1.3957	GADD45G	Regulating cell growth and repair
Macrophage	evm.TU.Chr1.3164	MIF	Immune response, inflammation regulation
	evm.TU.Chr3.5941	MRC1	Participate in the phagocytosis of immune cells

Table S2. Differential expression of key proteins in the liver of the black-spotted frog after exposure to 6PPD and 6PPD-Q.

Protein family	Protein ID	Symbol	Gene annotation
Glutathione	em.Chr2.4078	GSTM4	Detoxification of oxidative stress
Immunoglobulin	em.Chr1.3845	IGHE	Predict activation of antigen binding activity and immunoglobulin receptor binding activity
Catalase	em.Chr10.456	CAT	Antioxidant defense
Myosin	em.Chr8.1786	MYL9	Provide power for cell movement
Apolipoprotein	em.Chr4.5633	APOB	Cell signal recognition
Cathepsin	em.Chr13.595	CTSS	Immune response, cellular autophagy
Neutrophils	em.Chr8.2442	NCF4	Cellular immunity
Major histocompatibility complex	em.Chr7.3226	MR1	Antigen-presenting molecule
Cathepsin	em.Chr13.595	CTSS	Inflammation and immunity in the body
Lymphocyte	em.Chr5.1211	LY96	Inflammation, immune related
